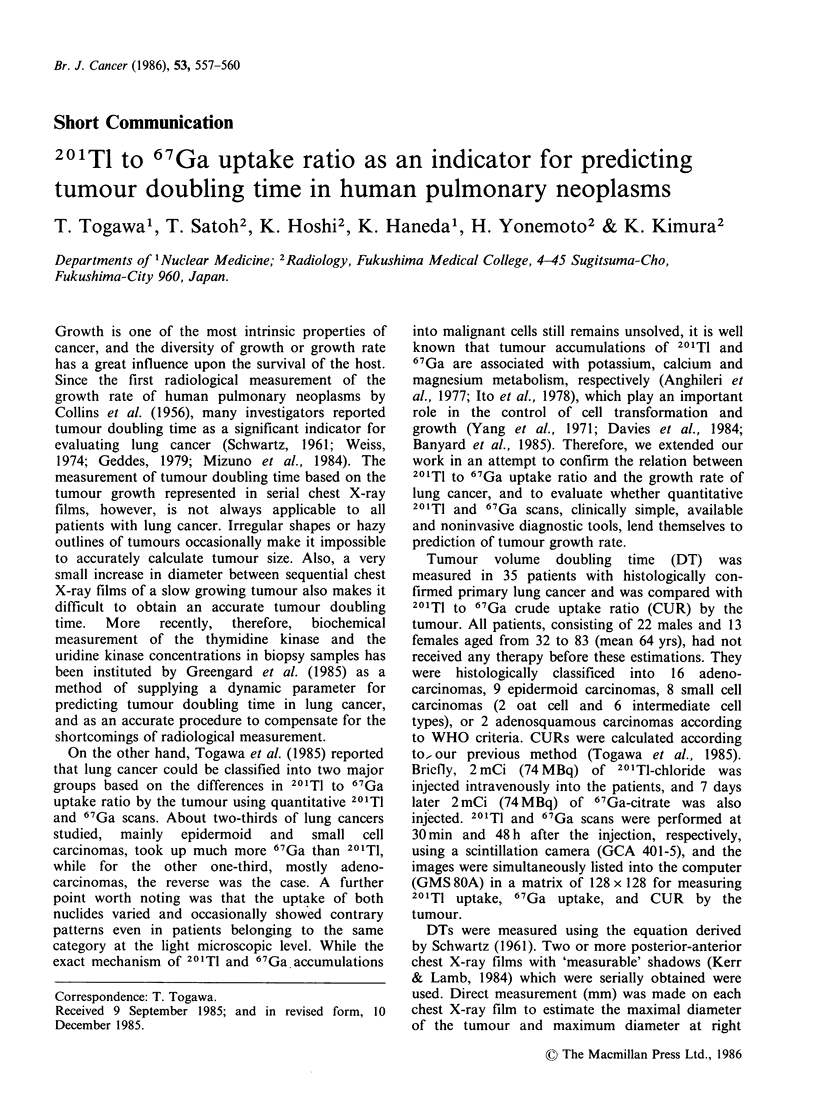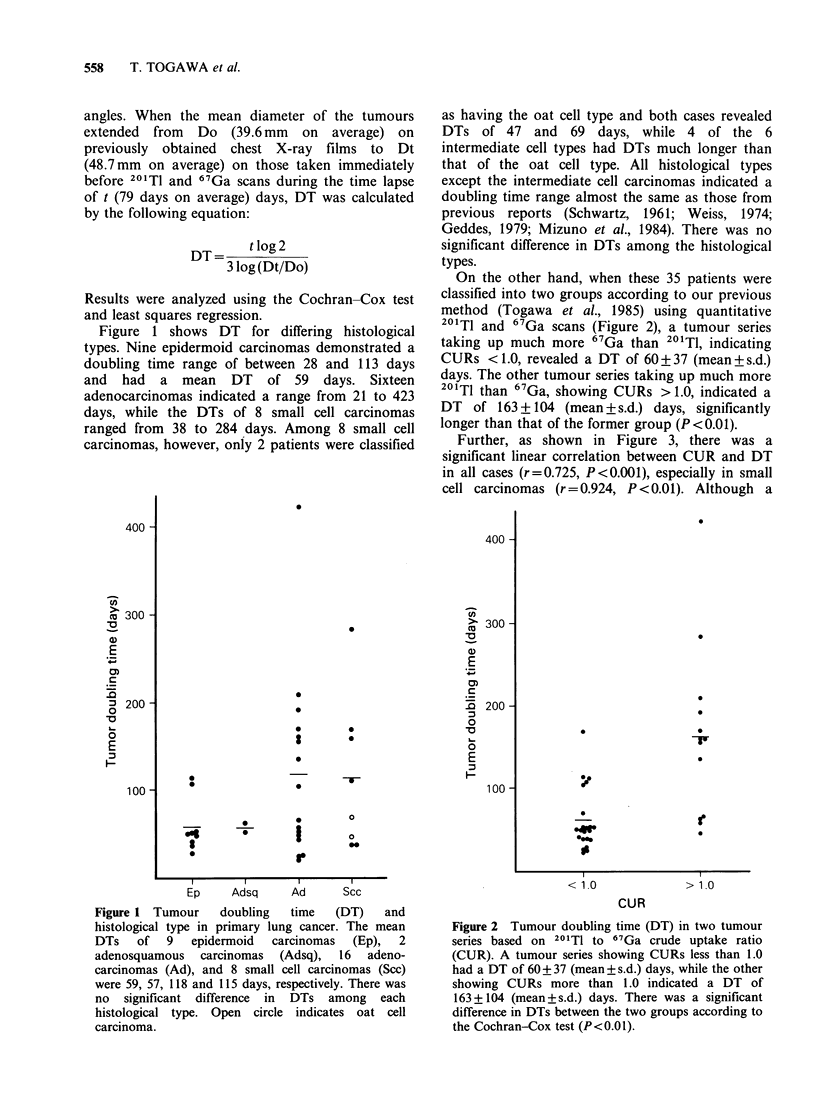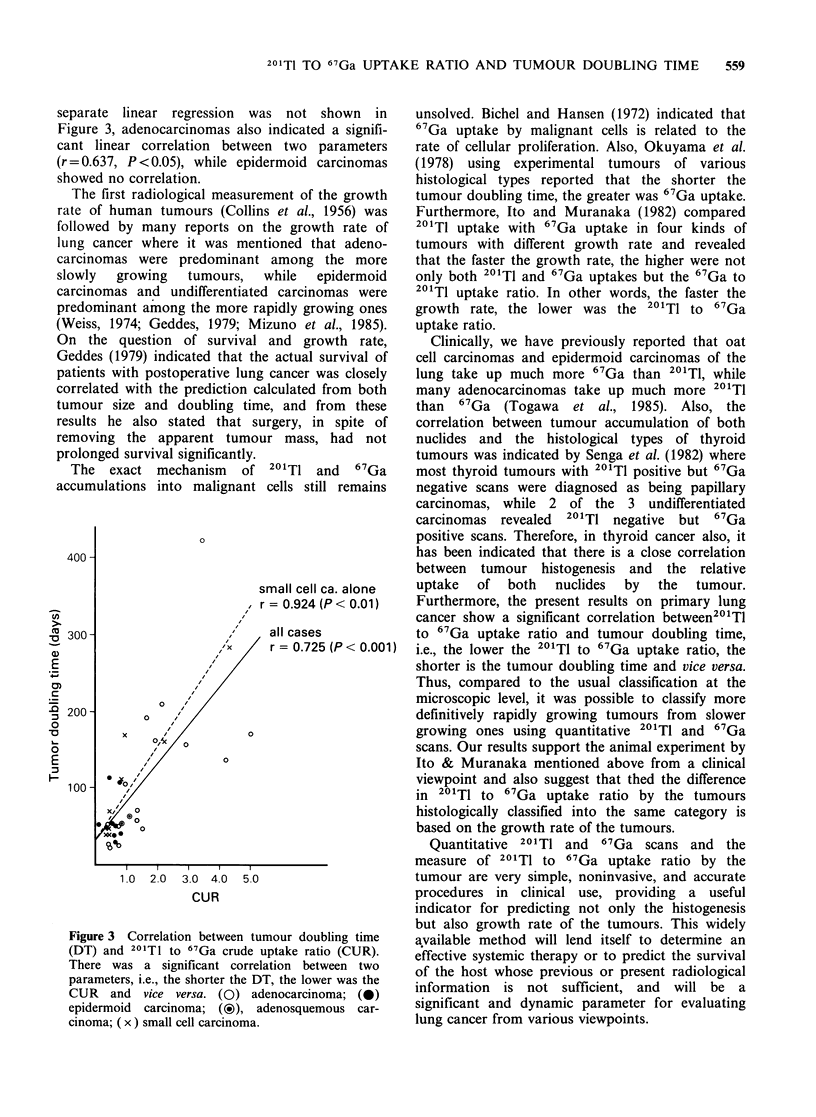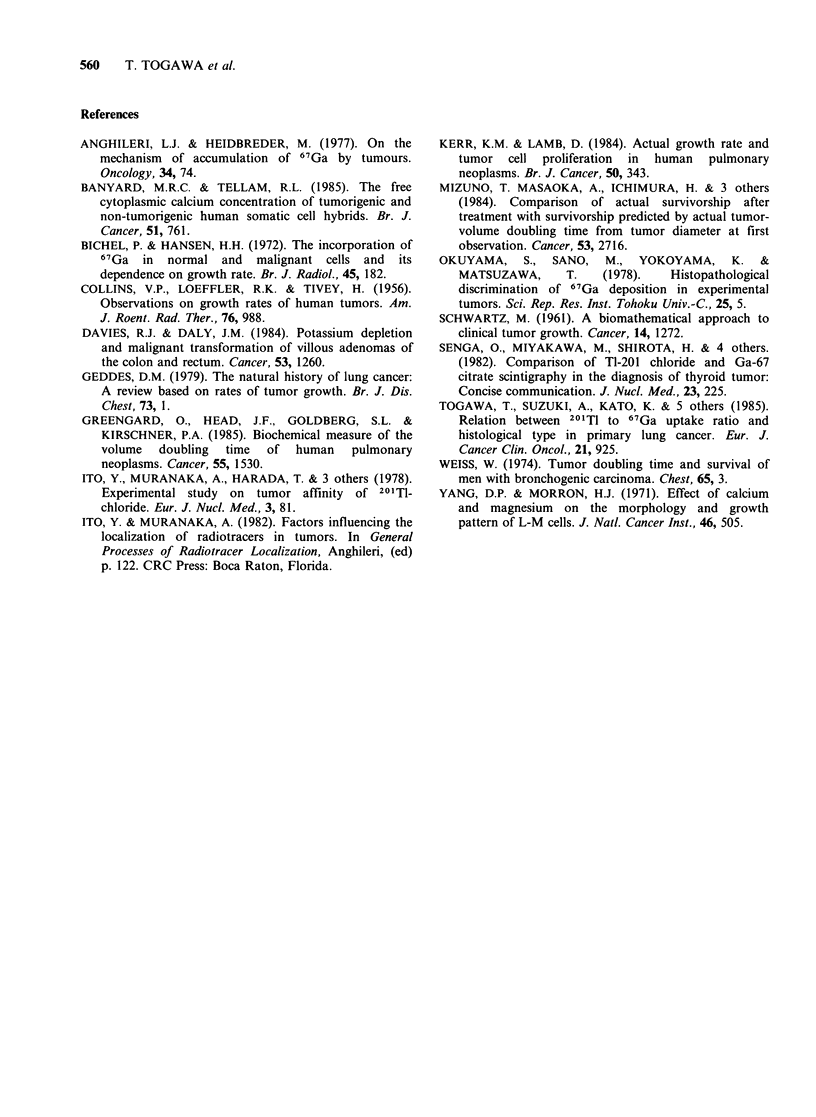# 201T1 to 67Ga uptake ratio as an indicator for predicting tumour doubling time in human pulmonary neoplasms.

**DOI:** 10.1038/bjc.1986.87

**Published:** 1986-04

**Authors:** T. Togawa, T. Satoh, K. Hoshi, K. Haneda, H. Yonemoto, K. Kimura


Br. J. Cancer (1986), 53, 557–560

### Short Communication

### $^{201}Tl$ to $^{67}Ga$ uptake ratio as an indicator for predicting tumour doubling time in human pulmonary neoplasms

T. Togawa[1], T. Satoh[2], K. Hoshi[2], K. Haneda[1], H. Yonemoto[2] & K. Kimura[2]

*Departments of [1]Nuclear Medicine; [2]Radiology, Fukushima Medical College, 4–45 Sugitsuma-Cho, Fukushima-City 960, Japan.*

Growth is one of the most intrinsic properties of cancer, and the diversity of growth or growth rate has a great influence upon the survival of the host. Since the first radiological measurement of the growth rate of human pulmonary neoplasms by Collins *et al.* (1956), many investigators reported tumour doubling time as a significant indicator for evaluating lung cancer (Schwartz, 1961; Weiss, 1974; Geddes, 1979; Mizuno *et al.*, 1984). The measurement of tumour doubling time based on the tumour growth represented in serial chest X-ray films, however, is not always applicable to all patients with lung cancer. Irregular shapes or hazy outlines of tumours occasionally make it impossible to accurately calculate tumour size. Also, a very small increase in diameter between sequential chest X-ray films of a slow growing tumour also makes it difficult to obtain an accurate tumour doubling time. More recently, therefore, biochemical measurement of the thymidine kinase and the uridine kinase concentrations in biopsy samples has been instituted by Greengard *et al.* (1985) as a method of supplying a dynamic parameter for predicting tumour doubling time in lung cancer, and as an accurate procedure to compensate for the shortcomings of radiological measurement.

On the other hand, Togawa *et al.* (1985) reported that lung cancer could be classified into two major groups based on the differences in $^{201}Tl$ to $^{67}Ga$ uptake ratio by the tumour using quantitative $^{201}Tl$ and $^{67}Ga$ scans. About two-thirds of lung cancers studied, mainly epidermoid and small cell carcinomas, took up much more $^{67}Ga$ than $^{201}Tl$, while for the other one-third, mostly adenocarcinomas, the reverse was the case. A further point worth noting was that the uptake of both nuclides varied and occasionally showed contrary patterns even in patients belonging to the same category at the light microscopic level. While the exact mechanism of $^{201}Tl$ and $^{67}Ga$ accumulations into malignant cells still remains unsolved, it is well known that tumour accumulations of $^{201}Tl$ and $^{67}Ga$ are associated with potassium, calcium and magnesium metabolism, respectively (Anghileri *et al.*, 1977; Ito *et al.*, 1978), which play an important role in the control of cell transformation and growth (Yang *et al.*, 1971; Davies *et al.*, 1984; Banyard *et al.*, 1985). Therefore, we extended our work in an attempt to confirm the relation between $^{201}Tl$ to $^{67}Ga$ uptake ratio and the growth rate of lung cancer, and to evaluate whether quantitative $^{201}Tl$ and $^{67}Ga$ scans, clinically simple, available and noninvasive diagnostic tools, lend themselves to prediction of tumour growth rate.

Tumour volume doubling time (DT) was measured in 35 patients with histologically confirmed primary lung cancer and was compared with $^{201}Tl$ to $^{67}Ga$ crude uptake ratio (CUR) by the tumour. All patients, consisting of 22 males and 13 females aged from 32 to 83 (mean 64 yrs), had not received any therapy before these estimations. They were histologically classificed into 16 adenocarcinomas, 9 epidermoid carcinomas, 8 small cell carcinomas (2 oat cell and 6 intermediate cell types), or 2 adenosquamous carcinomas according to WHO criteria. CURs were calculated according to our previous method (Togawa *et al.*, 1985). Briefly, 2 mCi (74 MBq) of $^{201}Tl$-chloride was injected intravenously into the patients, and 7 days later 2 mCi (74 MBq) of $^{67}Ga$-citrate was also injected. $^{201}Tl$ and $^{67}Ga$ scans were performed at 30 min and 48 h after the injection, respectively, using a scintillation camera (GCA 401-5), and the images were simultaneously listed into the computer (GMS 80A) in a matrix of $128 \times 128$ for measuring $^{201}Tl$ uptake, $^{67}Ga$ uptake, and CUR by the tumour.

DTs were measured using the equation derived by Schwartz (1961). Two or more posterior-anterior chest X-ray films with 'measurable' shadows (Kerr & Lamb, 1984) which were serially obtained were used. Direct measurement (mm) was made on each chest X-ray film to estimate the maximal diameter of the tumour and maximum diameter at right

Correspondence: T. Togawa.
Received 9 September 1985; and in revised form, 10 December 1985.

© The Macmillan Press Ltd., 1986

angles. When the mean diameter of the tumours extended from Do (39.6 mm on average) on previously obtained chest X-ray films to Dt (48.7 mm on average) on those taken immediately before $^{201}$Tl and $^{67}$Ga scans during the time lapse of $t$ (79 days on average) days, DT was calculated by the following equation:

$$DT = \frac{t \log 2}{3 \log(Dt/Do)}$$

Results were analyzed using the Cochran–Cox test and least squares regression.

Figure 1 shows DT for differing histological types. Nine epidermoid carcinomas demonstrated a doubling time range of between 28 and 113 days and had a mean DT of 59 days. Sixteen adenocarcinomas indicated a range from 21 to 423 days, while the DTs of 8 small cell carcinomas ranged from 38 to 284 days. Among 8 small cell carcinomas, however, only 2 patients were classified as having the oat cell type and both cases revealed DTs of 47 and 69 days, while 4 of the 6 intermediate cell types had DTs much longer than that of the oat cell type. All histological types except the intermediate cell carcinomas indicated a doubling time range almost the same as those from previous reports (Schwartz, 1961; Weiss, 1974; Geddes, 1979; Mizuno et al., 1984). There was no significant difference in DTs among the histological types.

On the other hand, when these 35 patients were classified into two groups according to our previous method (Togawa et al., 1985) using quantitative $^{201}$Tl and $^{67}$Ga scans (Figure 2), a tumour series taking up much more $^{67}$Ga than $^{201}$Tl, indicating CURs $<1.0$, revealed a DT of $60 \pm 37$ (mean $\pm$ s.d.) days. The other tumour series taking up much more $^{201}$Tl than $^{67}$Ga, showing CURs $>1.0$, indicated a DT of $163 \pm 104$ (mean $\pm$ s.d.) days, significantly longer than that of the former group ($P < 0.01$).

Further, as shown in Figure 3, there was a significant linear correlation between CUR and DT in all cases ($r = 0.725$, $P < 0.001$), especially in small cell carcinomas ($r = 0.924$, $P < 0.01$). Although a

**Figure 1** Tumour doubling time (DT) and histological type in primary lung cancer. The mean DTs of 9 epidermoid carcinomas (Ep), 2 adenosquamous carcinomas (Adsq), 16 adenocarcinomas (Ad), and 8 small cell carcinomas (Scc) were 59, 57, 118 and 115 days, respectively. There was no significant difference in DTs among each histological type. Open circle indicates oat cell carcinoma.

**Figure 2** Tumour doubling time (DT) in two tumour series based on $^{201}$Tl to $^{67}$Ga crude uptake ratio (CUR). A tumour series showing CURs less than 1.0 had a DT of $60 \pm 37$ (mean $\pm$ s.d.) days, while the other showing CURs more than 1.0 indicated a DT of $163 \pm 104$ (mean $\pm$ s.d.) days. There was a significant difference in DTs between the two groups according to the Cochran–Cox test ($P < 0.01$).

separate linear regression was not shown in Figure 3, adenocarcinomas also indicated a significant linear correlation between two parameters ($r = 0.637$, $P < 0.05$), while epidermoid carcinomas showed no correlation.

The first radiological measurement of the growth rate of human tumours (Collins *et al.*, 1956) was followed by many reports on the growth rate of lung cancer where it was mentioned that adenocarcinomas were predominant among the more slowly growing tumours, while epidermoid carcinomas and undifferentiated carcinomas were predominant among the more rapidly growing ones (Weiss, 1974; Geddes, 1979; Mizuno *et al.*, 1985). On the question of survival and growth rate, Geddes (1979) indicated that the actual survival of patients with postoperative lung cancer was closely correlated with the prediction calculated from both tumour size and doubling time, and from these results he also stated that surgery, in spite of removing the apparent tumour mass, had not prolonged survival significantly.

The exact mechanism of $^{201}$Tl and $^{67}$Ga accumulations into malignant cells still remains

**Figure 3** Correlation between tumour doubling time (DT) and $^{201}$Tl to $^{67}$Ga crude uptake ratio (CUR). There was a significant correlation between two parameters, i.e., the shorter the DT, the lower was the CUR and *vice versa*. (○) adenocarcinoma; (●) epidermoid carcinoma; (◉), adenosquemous carcinoma; (×) small cell carcinoma.

unsolved. Bichel and Hansen (1972) indicated that $^{67}$Ga uptake by malignant cells is related to the rate of cellular proliferation. Also, Okuyama *et al.* (1978) using experimental tumours of various histological types reported that the shorter the tumour doubling time, the greater was $^{67}$Ga uptake. Furthermore, Ito and Muranaka (1982) compared $^{201}$Tl uptake with $^{67}$Ga uptake in four kinds of tumours with different growth rate and revealed that the faster the growth rate, the higher were not only both $^{201}$Tl and $^{67}$Ga uptakes but the $^{67}$Ga to $^{201}$Tl uptake ratio. In other words, the faster the growth rate, the lower was the $^{201}$Tl to $^{67}$Ga uptake ratio.

Clinically, we have previously reported that oat cell carcinomas and epidermoid carcinomas of the lung take up much more $^{67}$Ga than $^{201}$Tl, while many adenocarcinomas take up much more $^{201}$Tl than $^{67}$Ga (Togawa *et al.*, 1985). Also, the correlation between tumour accumulation of both nuclides and the histological types of thyroid tumours was indicated by Senga *et al.* (1982) where most thyroid tumours with $^{201}$Tl positive but $^{67}$Ga negative scans were diagnosed as being papillary carcinomas, while 2 of the 3 undifferentiated carcinomas revealed $^{201}$Tl negative but $^{67}$Ga positive scans. Therefore, in thyroid cancer also, it has been indicated that there is a close correlation between tumour histogenesis and the relative uptake of both nuclides by the tumour. Furthermore, the present results on primary lung cancer show a significant correlation between$^{201}$Tl to $^{67}$Ga uptake ratio and tumour doubling time, i.e., the lower the $^{201}$Tl to $^{67}$Ga uptake ratio, the shorter is the tumour doubling time and *vice versa.* Thus, compared to the usual classification at the microscopic level, it was possible to classify more definitively rapidly growing tumours from slower growing ones using quantitative $^{201}$Tl and $^{67}$Ga scans. Our results support the animal experiment by Ito & Muranaka mentioned above from a clinical viewpoint and also suggest that thed the difference in $^{201}$Tl to $^{67}$Ga uptake ratio by the tumours histologically classified into the same category is based on the growth rate of the tumours.

Quantitative $^{201}$Tl and $^{67}$Ga scans and the measure of $^{201}$Tl to $^{67}$Ga uptake ratio by the tumour are very simple, noninvasive, and accurate procedures in clinical use, providing a useful indicator for predicting not only the histogenesis but also growth rate of the tumours. This widely available method will lend itself to determine an effective systemic therapy or to predict the survival of the host whose previous or present radiological information is not sufficient, and will be a significant and dynamic parameter for evaluating lung cancer from various viewpoints.

### References

ANGHILERI, L.J. & HEIDBREDER, M. (1977). On the mechanism of accumulation of $^{67}Ga$ by tumours. *Oncology*, **34**, 74.

BANYARD, M.R.C. & TELLAM, R.L. (1985). The free cytoplasmic calcium concentration of tumorigenic and non-tumorigenic human somatic cell hybrids. *Br. J. Cancer*, **51**, 761.

BICHEL, P. & HANSEN, H.H. (1972). The incorporation of $^{67}Ga$ in normal and malignant cells and its dependence on growth rate. *Br. J. Radiol.*, **45**, 182.

COLLINS, V.P., LOEFFLER, R.K. & TIVEY, H. (1956). Observations on growth rates of human tumors. *Am. J. Roent. Rad. Ther.*, **76**, 988.

DAVIES, R.J. & DALY, J.M. (1984). Potassium depletion and malignant transformation of villous adenomas of the colon and rectum. *Cancer*, **53**, 1260.

GEDDES, D.M. (1979). The natural history of lung cancer: A review based on rates of tumor growth. *Br. J. Dis. Chest*, **73**, 1.

GREENGARD, O., HEAD, J.F., GOLDBERG, S.L. & KIRSCHNER, P.A. (1985). Biochemical measure of the volume doubling time of human pulmonary neoplasms. *Cancer*, **55**, 1530.

ITO, Y., MURANAKA, A., HARADA, T. & 3 others (1978). Experimental study on tumor affinity of $^{201}Tl$-chloride. *Eur. J. Nucl. Med.*, **3**, 81.

ITO, Y. & MURANAKA, A. (1982). Factors influencing the localization of radiotracers in tumors. In *General Processes of Radiotracer Localization*, Anghileri, (ed) p. 122. CRC Press: Boca Raton, Florida.

KERR, K.M. & LAMB, D. (1984). Actual growth rate and tumor cell proliferation in human pulmonary neoplasms. *Br. J. Cancer*, **50**, 343.

MIZUNO, T. MASAOKA, A., ICHIMURA, H. & 3 others (1984). Comparison of actual survivorship after treatment with survivorship predicted by actual tumor-volume doubling time from tumor diameter at first observation. *Cancer*, **53**, 2716.

OKUYAMA, S., SANO, M., YOKOYAMA, K. & MATSUZAWA, T. (1978). Histopathological discrimination of $^{67}Ga$ deposition in experimental tumors. *Sci. Rep. Res. Inst. Tohoku Univ.-C.*, **25**, 5.

SCHWARTZ, M. (1961). A biomathematical approach to clinical tumor growth. *Cancer*, **14**, 1272.

SENGA, O., MIYAKAWA, M., SHIROTA, H. & 4 others. (1982). Comparison of Tl-201 chloride and Ga-67 citrate scintigraphy in the diagnosis of thyroid tumor: Concise communication. *J. Nucl. Med.*, **23**, 225.

TOGAWA, T., SUZUKI, A., KATO, K. & 5 others (1985). Relation between $^{201}Tl$ to $^{67}Ga$ uptake ratio and histological type in primary lung cancer. *Eur. J. Cancer Clin. Oncol.*, **21**, 925.

WEISS, W. (1974). Tumor doubling time and survival of men with bronchogenic carcinoma. *Chest*, **65**, 3.

YANG, D.P. & MORRON, H.J. (1971). Effect of calcium and magnesium on the morphology and growth pattern of L-M cells. *J. Natl. Cancer Inst.*, **46**, 505.